# Prolyl hydroxylase domain inhibitor is an effective pre-hospital pharmaceutical intervention for trauma and hemorrhagic shock

**DOI:** 10.1038/s41598-024-53945-w

**Published:** 2024-02-16

**Authors:** Xiaowu Wu, Andrew P. Cap, James A. Bynum, Tiffani C. Chance, Daniel N. Darlington, Michael A. Meledeo

**Affiliations:** 1 Blood and Shock Resuscitation, USA Army Institute of Surgical Research, 3698 Chambers Pass, Bldg 3610, JBSA Fort Sam Houston, TX 78234-7767 USA; 2 Department of Surgery, University of Texas Health Science Center at San Antonio, San Antonio, TX 78229 USA; 3 Department of Health and Human Services, Center for Devices and Radiological Health, Food and Drug Administration, Silver Spring, MD 20993 USA

**Keywords:** Drug discovery, Health care, Medical research, Signs and symptoms

## Abstract

Pre-hospital potentially preventable trauma related deaths are mainly due to hypoperfusion-induced tissue hypoxia leading to irreversible organ dysfunction at or near the point of injury or during transportation prior to receiving definitive therapy. The prolyl hydroxylase domain (PHD) is an oxygen sensor that regulates tissue adaptation to hypoxia by stabilizing hypoxia inducible factor (HIF). The benefit of PHD inhibitors (PHDi) in the treatment of anemia and lactatemia arises from HIF stabilization, which stimulates endogenous production of erythropoietin and activates lactate recycling through gluconeogenesis. The results of this study provide insight into the therapeutic roles of MK-8617, a pan-inhibitor of PHD-1, 2, and 3, in the mitigation of lactatemia in anesthetized rats with polytrauma and hemorrhagic shock. Additionally, in an anesthetized rat model of lethal decompensated hemorrhagic shock, acute administration of MK-8617 significantly improves one-hour survival and maintains survival at least until 4 h following limited resuscitation with whole blood (20% EBV) at one hour after hemorrhage. This study suggests that pharmaceutical interventions to inhibit prolyl hydroxylase activity can be used as a potential pre-hospital countermeasure for trauma and hemorrhage at or near the point of injury.

## Introduction

Trauma is the leading cause of death for Americans under the age of 46^1^. The data from both civilian and military trauma registries demonstrate that the majority of trauma deaths occur during the prehospital phase, and as many as over 20% of trauma deaths may be preventable if optimal trauma care is delivered near or at the point of injury^2–4^. Data from Operation Enduring Freedom (OEF) and Operation Iraqi Freedom (OIF) indicated that over 90% of potentially survivable deaths were due to hemorrhage^3,4^, which underscores the need for adequate resuscitation fluid and blood products at the point of injury. Although the current transportation time in combat casualty care is aligned with the “Golden Hour” policy to promote better outcomes by providing medical treatment, including resuscitation with blood products, within an hour of injury^5^, the benefits are only for those who can survive for the 60-min post-injury period. Additionally, an imbalance in the supply chain and demand for blood products is a common logistical burden for both civilian and military mass casualty events^6,7^ preventing hemostatic resuscitation at the point of injury or during the prehospital phase. Therefore, new approaches or countermeasures that can extend pre-hospital survival time may help bridge the gap and reduce the number of deaths prior to blood-based resuscitation.

Acute trauma and hemorrhagic shock ultimately lead to cell death and organ dysfunction resulting from tissue hypoperfusion and hypoxia, commonly known as shock. One of the compensatory mechanisms during hypoxic challenge occurs through stabilizing the level of hypoxia inducible factors (HIF), central mediators involved in facilitating cellular adaptation to oxygen deprivation^8^. HIFs are transcriptional heterodimer complexes consisting of an inducible and unstable α subunit (HIF-1α, HIF-2α, and HIF-3α) and a constitutively expressed and stable β subunit^9,10^. Prolyl hydroxylase domain-containing proteins 1–3 (PHD1–3) are the key oxygen sensors to regulate cellular levels of HIFα^9,11^. Under normal oxygen levels, HIFα is hydroxylated by prolyl hydroxylase using oxygen as a required co-substrate and subsequently degraded through a proteasomal proteolytic pathway^12^. HIFα is stabilized when prolyl hydroxylase is inactivated in hypoxic tissues, thereby activating the downstream genes to regulate cellular survival and adaptation to hypoxia^13,14^. Prolyl hydroxylase domain inhibitors (PHDi) have been developed to treat diseases that benefit from HIFα stabilization^15^. Since HIF-1α stimulates erythropoietin (EPO) production in the kidney and thus enhances marrow production and maturation of reticulocytes, PHDi have been used successfully to treat anemia in chronic renal failure^16,17^. Additionally, it has also been demonstrated that HIF-1α not only enhances glycolysis and lactate efflux from the cells but also increases the uptake of lactate for gluconeogenesis (Cori cycle), resulting in an acceleration of lactate clearance and amelioration of lactate acidosis under hypoxia^18,19^. Currently, several PHDi are in different phases of clinical trials for the treatment of anemia caused by chronic kidney disease^15^. MK-8617 is a pan-inhibitor of PHD1–3, originally invented as an oral treatment for anemia and tested across various species^20^. However, it is unknown whether the effects of MK-8617 will be beneficial for the treatment of shock resulting from trauma and hemorrhage.

Trauma with active bleeding can quickly lead to an acute global event of tissue hypoxia. Without prompt and sufficient volume replacement by any resuscitation fluid, tissue hypoxia will be further intensified with the development of decompensated hemorrhagic shock. Using various clinically relevant rodent models, this study sought to determine whether acute administration of PHDi can mitigate the rise of lactate in response to polytrauma and hemorrhage and improve the 1-h survival of lethal decompensated hemorrhagic shock prior to whole blood resuscitation.

## Results

PHDi (MK-8617 (MK)) was tested in three different scenarios of trauma and hemorrhage: (1) oral administration (by gavage) prior to polytrauma/hemorrhage; (2) intravenous administration at 20 min after trauma; and (3) intravenous administration at 20 min after lethal decompensated hemorrhage, followed by limited volume resuscitation with whole blood in surviving rats.

### Inhibition of prolyl hydroxylase prior to polytrauma/hemorrhage reduced lactate elevation

**Figure 1:**
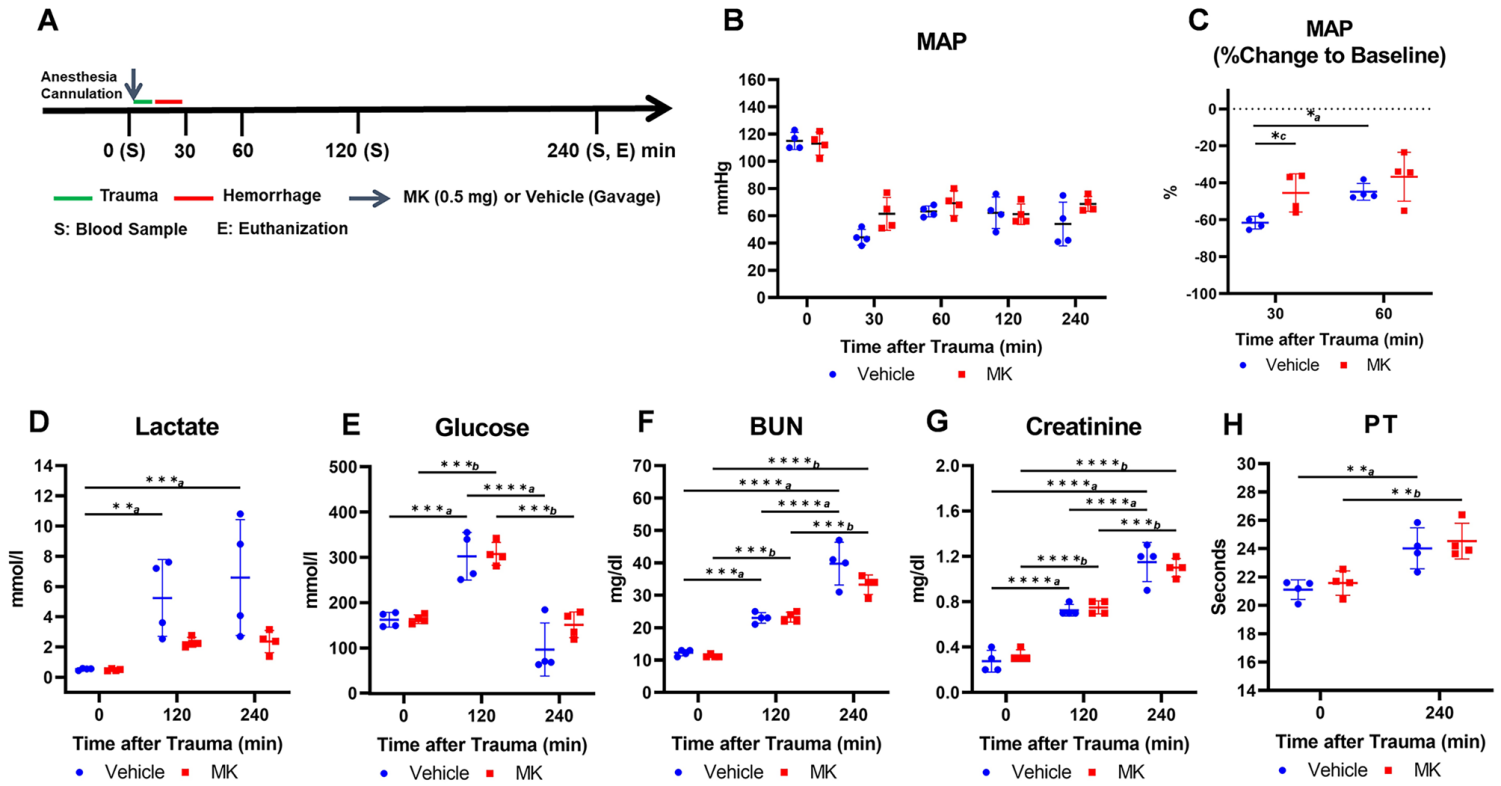
A pilot study of administration of MK-8617 (MK) prior to polytrauma/hemorrhage by gavage mitigated lactate increase. (**A**) Study schematic timeline; (**B**) sustained decline in MAP at 30, 60, 120, and 240 min after trauma followed by 40% hemorrhage. (**C**) A significant difference in MAP percent change versus baseline was observed between vehicle and MK 30 min after trauma. (**D**) Lactate levels were significantly elevated in vehicle-treated rats, but not in MK-treated rats at 120 and 240 min after trauma. (**E**) Glucose was significantly elevated at 120 min versus baseline after trauma and declined at 240 min compared to that at 120 min in both groups of the vehicle and MK. (**F**) BUN and (**G**) Creatinine were significantly and progressively elevated at 120 min and 240 min versus baseline after trauma in both groups; (**H**) PT significantly elevated at 240 min after trauma in both groups of the vehicle and MK versus baseline, and there was no significant difference between groups. Solid dot (blue): vehicle; solid square (red): MK; a: comparison among the groups of the vehicle; b: comparison among the groups of the MK; c: comparison between the groups of the vehicle and MK; *p < 0.05; **p < 0.01; ***p < 0.001; ****p < 0.0001. *MK* MK-8617; *MAP* mean arterial pressure; *PT* prothrombin time; *BUN* blood urea nitrogen.

This experiment, as illustrated in a schematic timeline (Fig. 1A), was a proof-of-concept study (n = 4 per group) to determine whether elevation of lactate after polytrauma followed by 40% hemorrhage could be mitigated if PHD was inactivated by PHDi (MK-8617, 0.5 mg) by oral administration prior to trauma. There was a sustained reduction in MAP at 30, 60, 120, and 240 min after trauma, followed by 40% hemorrhage (Fig. 1B). The average range of the percentage MAP reduction was between 40 and 60% from the baseline, and rats pre-treated with MK had a greater degree of early compensation in MAP than rats treated with vehicle (Fig. 1C), as shown by a significantly lower percentage change of MAP with MK than vehicle at 30 min after trauma. There was a significant elevation in plasma levels of lactate in rats treated with vehicle at 120 and 240 min after trauma, which trended to be mitigated by oral pre-treatment with MK (Fig. 1D). Glucose was significantly elevated at 120 min from the baseline and declined at 240 min after trauma, but no significant difference was found between the groups (Fig. 1E). Acute kidney injury and coagulopathy were characterized by a significant elevation of BUN and creatinine from the baseline (at 120 and 240 min after trauma, Fig. 1F,G) and a significant elongation of PT from the baseline (at 240 min after trauma, Fig. 1H) in both groups of vehicle and MK-treated rats, but there were no significant differences between the groups (see complete results of the biochemistry assay at Table S1).

### Inhibition of prolyl hydroxylase after polytrauma/hemorrhage reduced lactate elevation

**Figure 2:**
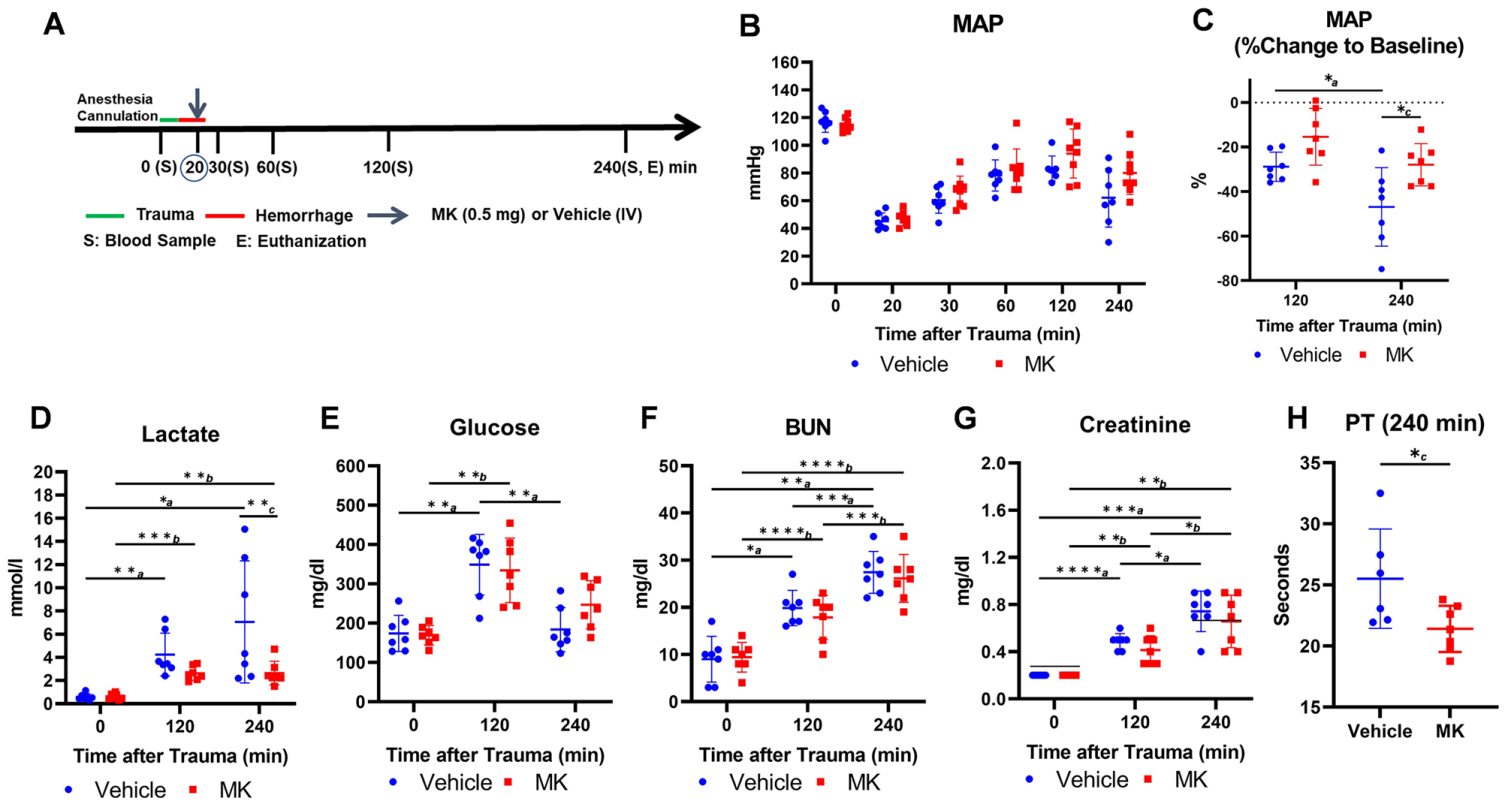
Intravenous administration of MK-8617 (MK) 20 min after polytrauma/hemorrhage significantly mitigated the rise of lactate. (**A**) Study schematic timeline; (**B**) sustained decline in MAP at 30, 60, 120, and 240 min after trauma followed by 40% hemorrhage. (**C**) A significant difference in MAP percent change versus baseline was observed between vehicle and MK 240 min after trauma; (**D**) lactate levels were significantly elevated versus baseline in both vehicle and MK-treated rats at 120 and 240 min after trauma; the lactate at 240 min was significantly lower in the rats of MK compared to the rats of vehicle. (**E**) Glucose was significantly elevated at 120 min compared to baseline and declined by 240 min compared to that at 120 min in both groups; no significant difference was found between the groups. (**F**) BUN and (**G**) Creatinine were significantly and progressively elevated at 120 min and 240 min after trauma in both groups; no significant difference was found between the groups. (**H**) PT was significantly reduced in rats treated with MK at 240 min after trauma compared to vehicle-treated rats. Solid dot (blue): vehicle; solid square (red): MK; a: comparison among the groups of the vehicle; b: comparison among the groups of the MK; c: comparison between the groups of the vehicle and MK; *p < 0.05; **p < 0.01; ***p < 0.001; ****p < 0.0001. *MK* MK-8617, *MAP* mean arterial pressure, *PT* prothrombin time, *BUN* blood urea nitrogen.

This experiment, as illustrated in a schematic timeline (Fig. 2A), was to determine whether elevation of lactate after polytrauma followed by 40% hemorrhage could be mitigated by early intravenous administration of MK (0.5 mg) after trauma. In this model (n = 7 per group), MK given 20 min after trauma led to a better compensation in blood pressure versus vehicle, as shown by the significantly lower percentage change in MAP at 240 min after trauma (Fig. 2B,C). Consistent with pre-treatment, lactate was significantly elevated at 120 and 240 min after trauma, which was mitigated in rats treated with MK (statistically significant at 240 min between the groups, Fig. 2D). Again, the glucose was significantly elevated at 120 min and declined by 240 min after trauma, but no significant difference was found between the groups (Fig. 2E). There was also no significant difference in elevation of BUN or creatinine between the groups at 120 and 240 min after trauma (Fig. 2F,G). The prothrombin time at 240 min was significantly reduced in rats treated with MK (Fig. 2H) (see complete results of the biochemistry assay at Table S2).

### HIF-1α and the expression of HIF-1α downstream regulated genes in the tissues after polytrauma/hemorrhage

**Figure 3:**
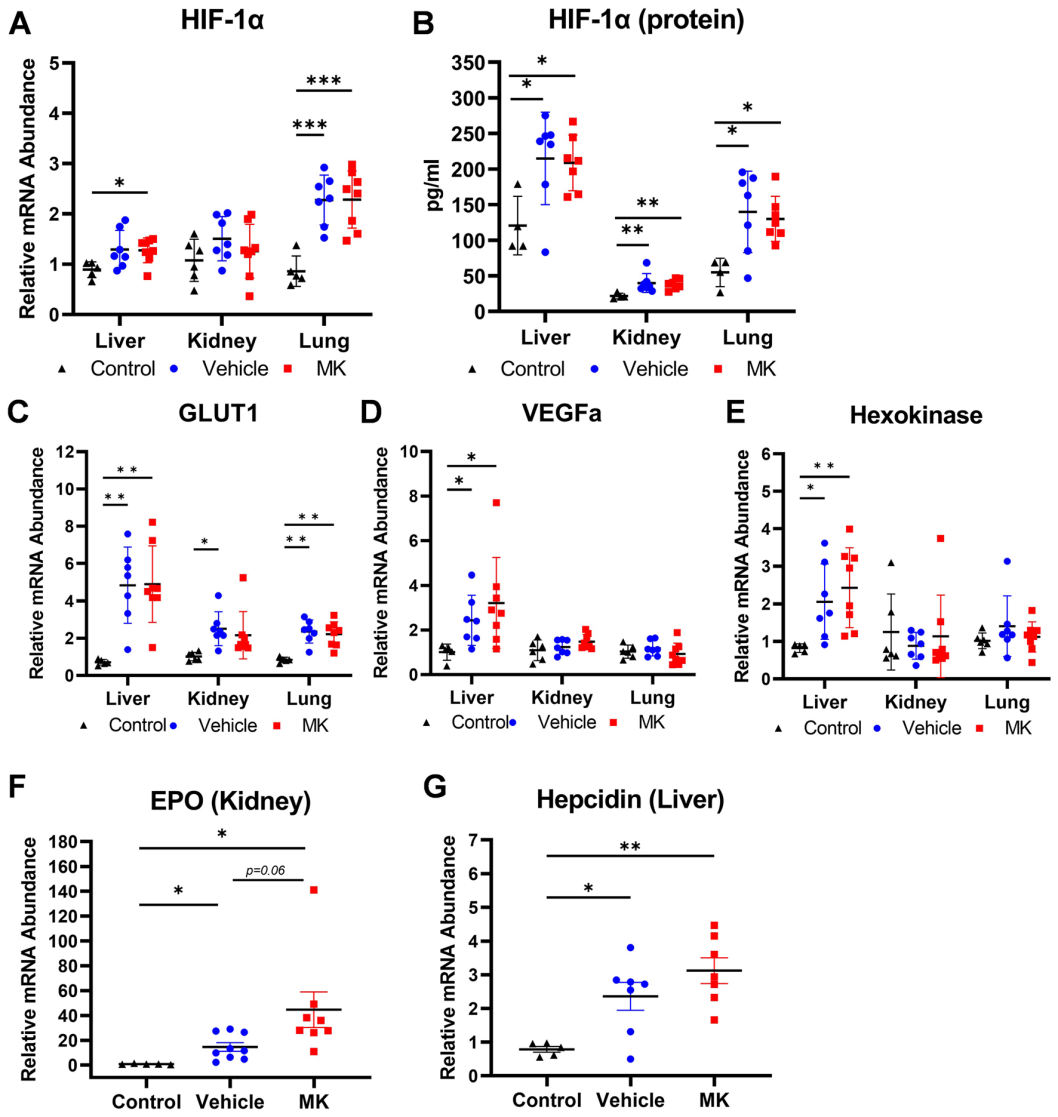
HIF-1α mRNA/protein and targeted downstream gene expression in the tissues (liver, kidney and lung) collected at 240 min after polytrauma/hemorrhage. (**A**) Gene expression and (**B**) protein expression of HIF-1α in liver, kidney, and lung at 240 min after polytrauma/hemorrhage; HIF-1α targeted downstream gene expression: (**C**) GLUT1, (**D**) VEGFa, and (**E**) hexokinase, in the liver, kidney, and lung among the groups of sham control, vehicle, and MK; (**F**) significant elevation of EPO gene expression in the kidney of both vehicle and MK-treated groups compared to sham control. EPO gene expression trended higher in MK-treated rats compared to vehicle (p = 0.06); (**G**) significant elevation of Hepcidin gene expression in the liver of both vehicle and MK-treated groups compared sham control. Solid triangle (black): control; Solid dot (blue): vehicle; solid square (red): MK; *p < 0.05; **p < 0.01; ***p < 0.001. *HIF-1α* hypoxia-inducible factor-1 alpha, *GLUT1* glucose transporter protein type 1, *VEGFa* vascular endothelial growth factor A, *EPO* erythropoietin.

Among the tissues collected after euthanasia at 240 min, HIF-1α mRNA was slightly elevated in the livers and kidneys and strongly elevated in the lungs in all rats of both groups compared to the sham control (Fig. 3A). There was a significant elevation of HIF-1α protein in the livers, kidneys, and lungs of all rats compared to the sham control, but no significant difference was found between the groups of vehicle and MK (Fig. 3B). Regardless of the treatment, the HIF-1α downstream genes GLUT1, hexokinase, and VEGFa were consistently upregulated in the livers, but only GLUT1 was elevated in the kidneys and lungs (Fig. 3C–E). For the genes associated with HIF-1α-induced hematopoiesis, EPO gene expression in the kidney was significantly elevated in both groups, and a higher level of EPO gene expression was found in PHDi-treated rats (Fig. 3F). The hepcidin gene expression was elevated significantly in both vehicle- and PHDi-treated rats, but no significant difference was found between the groups (Fig. 3G).

### Inhibition of prolyl hydroxylase significantly extended the survival time in rats with lethal decompensated shock

**Figure 4:**
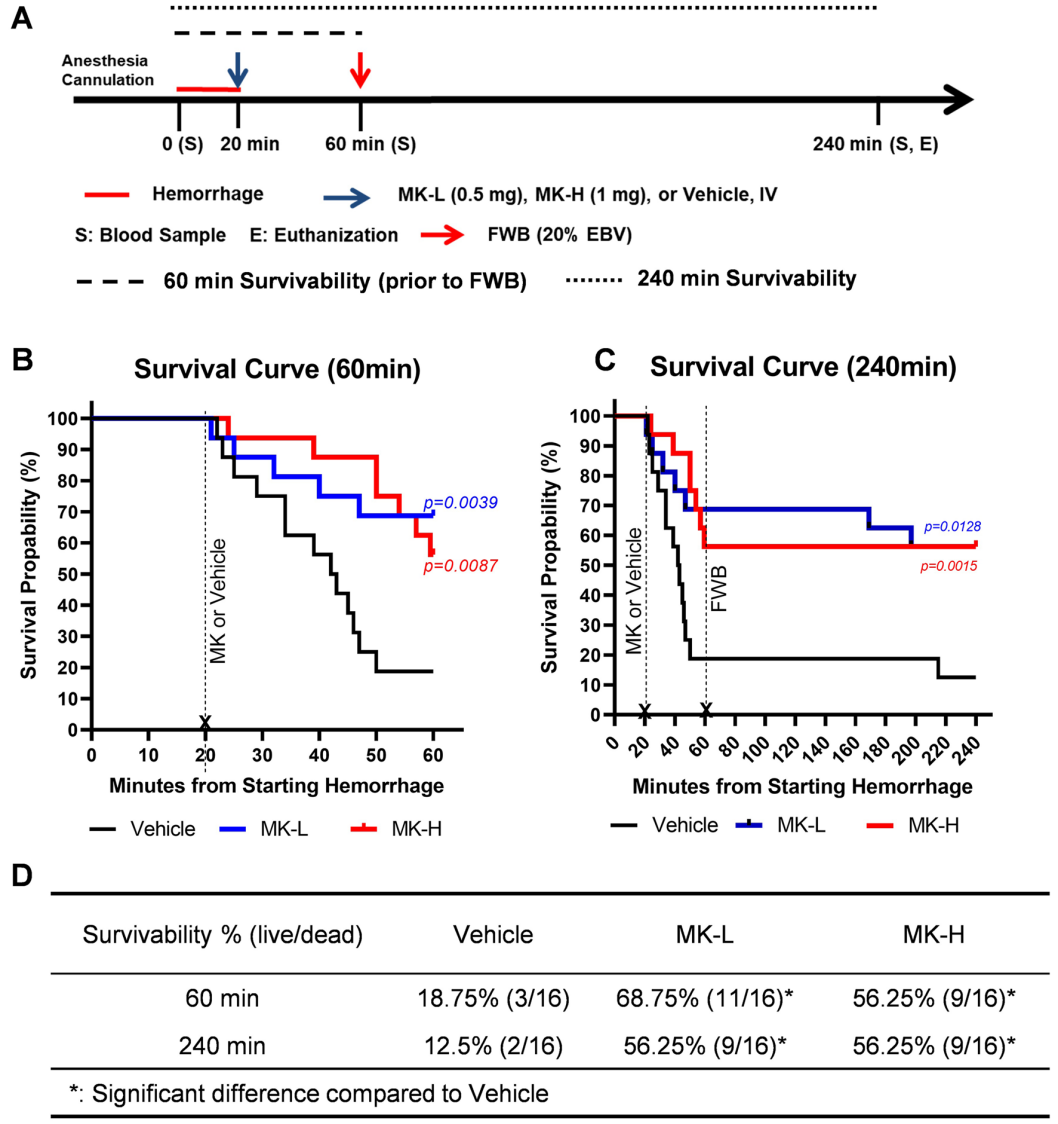
Administration of MK-8617 (MK) improves survivability in lethal decompensated hemorrhagic shock. (**A**) Study schematic timeline; (**B**) Kaplan–Meier analysis of rats treated with vehicle (black), MK-L (blue), or MK-H (red) 60 min after initiating hemorrhage and prior to FWB resuscitation, and (**C**) for 240 min or 180 min after FWB resuscitation. The survivability (Log-rank test) was significantly higher in rats treated with MK-L or MK-H compared to rats treated with vehicle at 60 and 240 min after hemorrhage; no significant difference was found between MK-L and MK-H. (**D**) The survivability rate for vehicle, MK-L, and MK-H treatments at 60 min and 240 min after hemorrhage showed significant differences comparing MK-L or MK-H to vehicle (Chi-square or Fisher’s exact test); no significant difference was found between MK-L and MK-H. *p < 0.05. *MK-L* MK-8617 lose dose, *MK-H* MK-8617 high dose.

A 65% hemorrhage rat model was lethal before 60 min without resuscitative intervention (Fig. S1). This experiment, as illustrated in a schematic timeline (Fig. 4A), was to determine whether acute intravenous administration of 0.5 mg MK-8617 (MK-L) or 1 mg MK-8617 (MK-H) could extend the survival time to 60 min, improve outcomes, and accommodate limited volume fresh whole blood (FWB) resuscitation. There was a significant improvement in survivability in MK-treated rats compared to vehicle at 60 min [MK-L (68.8%), MK-H (56.3%) vs. vehicle (18.8%)]. Comparing to the rats treated with vehicle, the rats receiving the treatment of MK-L or MK-H at 20 min from starting the hemorrhage significantly improved their survivability prior to whole blood resuscitation at 60 min after the hemorrhage, as shown in the Kaplan–Meier plots with log-rank tests (Fig. 4B) and 60-min-survivability analysis with Fisher’s exact test (Fig. 4D). Among the surviving rats that received limited volume whole blood resuscitation at 60 min, 9 of 11 MK-L, 9 of 9 MK-H and 2 of 3 vehicle-treated rats survived for 3 h following FWB resuscitation, and the 240 min survival rate (56.3%) for both MK-L and MK-H was significantly higher compared to vehicle (12.5%) (Fig. 4C,D). However, there was no significant difference in survivability between the groups of MK-L and MK-H.

### Acute administration of prolyl hydroxylase inhibitor followed by whole blood resuscitation revealed a dose-dependent improvement of the outcome in rats with lethal decompensated shock

**Figure 5:**
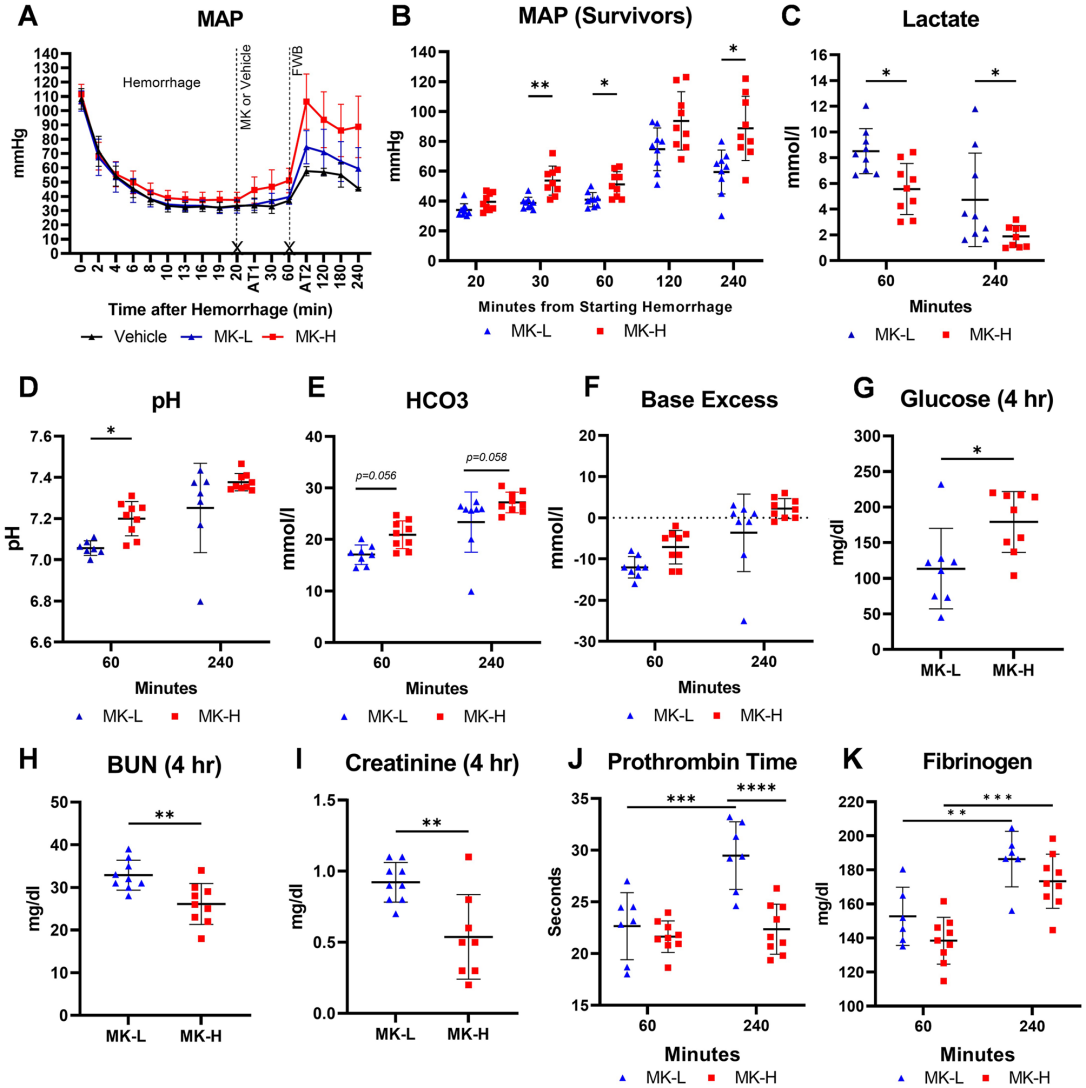
Administration of MK-8617 (MK) results in a dose-dependent improvement of outcomes in lethal decompensated hemorrhagic shock. (**A**) There was a sustained decline in MAP in response to hemorrhage prior to FWB resuscitation at 60 min, and FWB resuscitation partially restored MAP at least for 3 h after resuscitation in surviving animals; the MAP in rats of MK-H showed better compensation prior to FWB resuscitation and was maintained at the highest level of MAP compared to that in rats of MK-L and the vehicle. (**B**) MAP was significantly higher in rats of MK-H compared to rats of MK-L at 30, 60, and 240 min after hemorrhage. (**C**) Lactate was significantly lower in rats of MK-H compared to rats of MK-L at 60 and 240 min after hemorrhage. (**D**) pH was significantly higher in rats of MK-H compared to rats of MK-L at 60 min after hemorrhage. (**E**) HCO3 was trending higher in MK-H compared to rats of MK-L at 60 and 240 min after trauma. (**F**) There was no significant difference in base excess between the groups of MK-H and MK-L. (**G**) Glucose was significantly higher in rats of MK-H compared to rats of MK-L at 240 min after hemorrhage. (**H**) BUN and (**I**) Creatinine were significantly lower in rats of MK-H compared to rats of MK-L at 240 min after hemorrhage. (**J**) Improvement in coagulopathy with MK-H treatment compared to MK-L was observed by a significant reduction of PT at 180 min after FWB resuscitation. (**K**) FWB resuscitation improved the levels of fibrinogen in both groups of MK, but there was no significant difference between the groups. Solid dot (blue): MK-L; solid square (red): MK-H; *p < 0.05; **p < 0.01; ***p < 0.001; ****p < 0.0001. *MK-L* MK-8617 lose dose, *MK-H* MK-8617 high dose.

There was a significant improvement in MAP in rats of MK-H compared to MK-L at 30 min prior to resuscitation (60 min), and three hours after FWB resuscitation (240 min) (Fig. 5A), and the MAP in rats of MK-H was significantly higher than that in rats of MK-L at 30, 60, and 240 min between groups (Fig. 5B). Comparing the metabolic outcome of the rats of MK-H to the rats of MK-L, there was a significant reduction in the rise of lactate (Fig. 5C) and a significant or trend to higher levels of pH, bicarbonate, and base excess in the MK-H group versus MK-L (Fig. 5D–F, respectively) at 60 min (prior to FWB resuscitation) and/or 240 min (3 h after FWB resuscitation) after hemorrhage. The glucose level at 240 min was restored in rats of MK-H (179 ± 43 mg/dl vs. 184 ± 32 mg/dl at baseline), which was significantly higher than that of rats in the MK-L group at 240 min (104 ± 36 mg/dl, p < 0.05) (Fig. 5G, and complete biochemistry data at Table S3). In surviving rats receiving FWB resuscitation, the signs of acute kidney injury (blood urea nitrogen (BUN) and creatinine levels) were significantly mitigated (Fig. 5H,I), and hemostasis (marked by PT) was significantly improved in rats of the MK-H group (Fig. 5J) without a difference in the levels of fibrinogen between the groups (Fig. 5K). RBC counts, along with hemoglobin and hematocrit, were not significantly different between groups of MK-L and MK-H (Table S4).

## Discussion

We demonstrated a beneficial therapeutic effect of MK-8617, a pan-PHD inhibitor, in the acute treatment of trauma and hemorrhage. In a rodent model of polytrauma followed by 40% hemorrhage, administration of MK-8617 either prior to trauma or at 20 min after trauma resulted in a reduction in lactate elevation and an improvement in hemodynamic compensation. In a rodent model of lethal decompensated hemorrhagic shock, administration of MK-8617 at 20 min from initiation of hemorrhage resulted in a significantly higher survival rate at 60 min, and a dose-dependent improvement of the outcomes was observed in surviving rats at 60 min, immediately prior to FWB resuscitation, and at 3 h after FWB resuscitation. For the first time, the current study evaluates the outcomes of PHDi in rodent models that mimic common scenarios related to battlefield trauma, and the results suggest a potential application of PHDi as a novel pharmaceutical countermeasure for trauma and hemorrhage at the point of injury or during the prehospital phase.

The revealed benefit of PHD inhibition in trauma and hemorrhage was to reduce lactate, as shown in the animals treated with MK-8617 before or after trauma. A previous study demonstrated that inactivation of PHD2 systemically or in the liver increased lactate clearance after treadmill exercise and thereby improved endurance and extended the time to exhaustion^18^. That study also suggested that inhibiting PHD2 enhances the uptake of lactate for gluconeogenesis through the Cori cycle^18^. The lactate level is a sensitive indicator of tissue hypoperfusion, and monitoring lactate clearance is used as an early indicator for the overall adequacy of resuscitation in trauma patients^21–24^, as well as having a predictive value in other critical conditions such as sepsis^25^ and cardiac arrest^26^. Lactatemia is associated with multiple organ failure and eventually leads to cardiac arrest if not adequately corrected^27,28^. In addition to the pilot proof-of-concept study showing a mitigation of trauma/hemorrhage-induced lactate elevation by pre-treatment of PHDi, we also demonstrated that a single dose of PHDi administered intravenously early after trauma attenuated the rise of lactate. Moreover, in rats with lethal decompensated hemorrhagic shock, the improvement in survival at 60 min was also considered to be a result of the corresponding reduction in lactate elevation, whereas a dose-dependent reduction in lactate and accordingly an increase in glucose levels were consistent with previous mechanistic studies suggesting that PHDi facilitates lactate recycling through gluconeogenesis. Although the glucose levels were not significantly different between the vehicle and MK-8617 groups in the model of polytrauma/hemorrhage, there was a negative linear correlation between lactate and glucose at 4 h in the rats treated with MK-8617 (r^2^ = 0.57, p = 0.052) but not in vehicle-treated rats (Fig. 2S). Certainly, the mitigation of the levels of lactate during hemorrhagic shock is also associated with improvements in blood pressure compensation, as shown by the higher MAP in MK-treated rats. PHDi has not been studied in any model of cardiovascular compensation during hypovolemic hypotension, but PHDi may augment compensatory mechanisms through further stabilizing the HIF, a key player in the regulation of blood pressure, by augmenting sympathetic adrenergic activity through enhancement of carotid chemosensory reflex^29^, improving cardiac contractility and cardiac output during myocardial ischemia or ischemia reperfusion injury^30–32^, and regulating the peripheral vascular tone and controlling peripheral vascular resistance^33,34^.

PHDi have undergone a clinical trial for the treatment of anemia in chronic kidney disease due to their actions in upregulating EPO expression in the kidney and downregulating hepcidin in the liver^22^. Compared to chronic kidney disease, acute trauma and hemorrhage is a more complex and multifactorial event. As shown in these results, the expression of both EPO and hepcidin was elevated at 4 h after trauma and hemorrhage and trended higher in MK-treated rats. Since the expression of EPO and hepcidin could be upregulated by multiple factors (such as proinflammatory cytokines) after trauma and hemorrhage^35,36^, the tendency toward higher expression of EPO and hepcidin in MK-treated rats may not be solely the result of HIF-1α by MK treatment. The animal model of polytrauma and hemorrhage led to tissue hypoxia^24^, which was not only characterized by the changes in blood biochemistry but also supported by the elevation of HIF-1α protein levels in the tissues. However, the higher levels of HIF-1α were not observed as expected in the tissues of MK-treated rats as compared to vehicle controls at 4 h after trauma. Accordingly, the expression of genes downstream of HIF, such as GLUT1, Hexokinase, or VEGFα, increased in various tissues of both vehicle- and MK-treated rats, but there were no significant differences between the groups. Certainly, those downstream genes might not be solely regulated by HIF-1α in this complex system. It is notable that the HIF-1α protein and downstream gene expression were only measured hours after treatment in the current study, and the significant effect of MK-8617 may be sustainably observed despite the fact that the half-life of MK-8617 was reported to be up to 9 h in healthy rats^37^. The results from that survival study with a controlled model of hemorrhagic shock suggested a dose-dependent benefit of MK-8617 in the reduction of lactate and improvement of hemodynamics, hemostasis, and acute kidney injury, and dosing optimization of MK is warranted for future studies.

The PHD inhibitors recently developed by multiple pharmaceutic companies for the treatment of renal anemia are all oral-based formulations^15,38^, and there are currently no liquid formulations that can be directly used when the oral route is inaccessible (e.g., at or near the point of injury for acute trauma). Generally, the oral dosing schedule that has been used in multiple clinical trials to treat renal anemia has not led to additional serious safety complications as compared to traditional treatment using erythropoiesis-stimulating agent (ESA)^39–41^, but an optimal dosing regimen of intravenously administered PHDi needs to be determined for acute trauma and hemorrhage to provide assurance that no critical side effects arise from sustained levels of HIF. In rodent studies, it has been previously reported that inhibition of HIF-1α ameliorates acute lung injury in trauma and hemorrhagic shock^42^. In trauma and hemorrhagic shock, ischemia and hypoxia lead to a stabilization of HIF-1α to mediate not only anti-inflammatory and reparative signals but also pro-inflammatory cytokines that could further induce HIF activity through positive feedback and thereby potentially exacerbate the severity of inflammatory lung injury^43,44^. In this study, not surprisingly, HIF-1α gene expression was elevated alongside the downstream target genes in the lung after trauma and hemorrhage. Therefore, administration of a PHD inhibitor may augment the stabilization of HIF-1α in response to ischemic hypoxia and potentially lead to off-target side effects such as secondary inflammatory organ injury (lung or otherwise). We previously demonstrated that multiple organ failure developed in this model, including acute lung injury and acute kidney injury^45,46^; however, as shown in this current study, MK-8617 treatment improved these outcomes. Transient administration of PHDi has also demonstrated safety in other disease treatments to regulate metabolism^18,47^, angiogenesis^48,49^, and cellular survival^50,51^, despite the fact that some adverse effects in chronic administration were reported previously, such as cardiomyopathy, hepatic steatosis, and polycythemia^52–54^.

It has been demonstrated in both civilian and military trauma populations that human whole blood is an efficient resuscitation fluid for severe trauma and hemorrhage; whole blood provides all components in one product and achieves better outcomes than component therapy in restoration of blood volume, oxygen delivery, and mitigation of acute traumatic coagulopathy^55–59^. The benefits of FWB were also demonstrated in rodent models of polytrauma and hemorrhage: a limited volume of FWB restored hemodynamic and metabolic function and mitigated tissue hypoxia^24,60^. This study showed that acute administration of MK-8617 in lethal decompensated hemorrhagic shock extended survival time to 60 min without adequate fluid resuscitation. Aligned with the current “golden hour” policy^5^, this study used a limited volume (20% EBV) FWB resuscitation for survivors 60 min after starting hemorrhage, further extending survivability. This discovery expands the ability to successfully reduce mortality within the “golden hour” and reduces the logistic burden of requiring blood-based resuscitation at the point of injury. At 3 h after FWB transfusion, better outcomes were maintained in the high-dose PHDi group compared to the low-dose group, suggesting that MK-8617 may improve FWB efficacy in the treatment of hemorrhagic shock.

Although there has been a lot of innovation in trauma care, there are serious limitations in developing effective pharmaceutical interventions for immediate use at the point of injury. There have been a few reported studies demonstrating the beneficial effects of PHD inhibitors in improving survival in lethal models of lactic acidosis^18^, acute respiratory distress syndrome (ARDS)^61^, and lethal dose radiation^50^. MK-8617 has been shown to stabilize and increase HIF levels by inhibiting PHD1, 2, and 3 in various species, including rodents^37^. This study provided evidence of the survival benefit of using this particular pan-PHD inhibitor as early as 20 min when over 60% of the blood was removed from the rodent. Since this study was not primarily designed for mechanistic exploration, the acute impact of PHDi on survival signaling was not fully elucidated in trauma and hemorrhagic shock. HIF-1α and HIF-2α are the two isoforms of HIF, but only HIF-1α was measured in this study. How the two HIF isoforms respond to PHDi treatment and how they work alone or together is not well characterized in trauma and hemorrhagic shock^62^. Additionally, this study was only conducted in male rats, although the results are expected to be applicable to female subjects as well, despite sex hormones (e.g., estrogen) potentially contributing to various outcomes in trauma and hemorrhagic shock.

### Conclusion

Inhibition of prolyl hydroxylase mitigates the lactate elevation in trauma/hemorrhage-induced hypoxia and extends the survival time of decompensated hemorrhagic shock in rats. For those rats that survive to 60 min and receive limited volume FWB resuscitation, there is also a dose-dependent benefit of PHDi in the improvement of hemodynamic compensation and mitigation of lactatemia, acute kidney injury, and coagulopathy. The results provide insight into the therapeutic role of PHDi in metabolic regulation under hemorrhagic shock, with supporting evidence that inhibition of prolyl hydroxylase activity increases lactate recycling during hyperlactatemia. This study suggests that inhibition of prolyl hydroxylase can be used as a potential pre-hospital countermeasure in the treatment of trauma and hemorrhage prior to having access to blood products. However, the dose and time of treatment need to be further optimized in the future.

## Materials and methods

### Animal use

Male rats in the weight range of 370–430 g (Sprague–Dawley from Charles Rivers, www.criver.com) were used. The light/dark cycle was 12 h of light and 12 h of darkness. Food [Laboratory Rodent Diet 5001 (www.LabDiet.com)] and water were given ad libitum. Animal experiments, including all surgical procedures, were performed under anesthesia using 1.5–2.5% Isoflurane (Forane, Baxter, US) with 100% oxygen.

### Rat model of polytrauma with hemorrhagic shock

This animal model was performed in Sprague–Dawley rats and previously demonstrated to recapitulate the pathologic changes of trauma and hemorrhagic shock in humans^63–65^. Briefly, polytrauma was performed in anesthetized rats, including sequential injuries comprised of laparotomy, crush injury on the small intestine and liver, bone fracture at the right femur, and crush injury on skeletal muscle. A fixed-volume/pressure-controlled hemorrhage was started immediately after tissue injury by repeatedly withdrawing blood via the femoral vein to maintain mean arterial blood pressure at 40 mmHg until 40% of the estimated blood volume (EBV: 6% of body weight + 0.77^66^) was removed. The 500 µl MK-8617 [MK, 1 mg/ml in 5% DMSO (25 µl), 10% PEG-400 (50 µl), and 85% normal saline (425 µl)] or vehicle (dissolvent only) was either given by gavage prior to trauma (n = 4 per group) or injected intravenously at 20 min after starting the trauma (n = 7 per group). Blood samples were taken at baseline, 60 min (only in intravenous administration groups), 120 min, and 240 min after trauma (prior to euthanasia), and the volume of the basal- and 120-min-blood samples was counted as a part of the total hemorrhage. Surviving rats were euthanized at 240 min post-trauma. The rats in sham control (n = 4–6) underwent the same procedure of cannulation and blood sampling except for hemorrhage and trauma.

### Rat model of lethal decompensated hemorrhagic shock

The fixed-volume hemorrhage (65% of EBV) was performed in anesthetized rats. Briefly, the initial 40% of total calculated hemorrhage was removed within the first 2 min from the femoral artery, followed by repeatedly withdrawing 1.5 ml of the blood every 2 min until 10 min from the beginning hemorrhage and 1 ml of blood every 3 min thereafter through the femoral vein until the targeted volume of hemorrhage was achieved. Blood samples taken at baseline (1 ml) and 60 min (0.5 ml) were counted as a part of the total amount of hemorrhage. At 20 min immediately prior to completion of hemorrhage, the rats were treated with 500 µl MK-8617 at 1 mg/ml (0.5 mg, MK-L), 2 mg/ml (1 mg, MK-H), or vehicle (n = 16 each) intravenously. At one hour after hemorrhage, the surviving rats were resuscitated by a limited volume of whole blood (20% EBV, equivalent to 2 units of whole blood in humans) using collected shed blood from the first 2 min of hemorrhage and then euthanized at 4 h (3 h after resuscitation).

### Measurements

The hemodynamics [mean arterial blood pressure (MAP) and heart rate (HR)] were recorded through femoral artery cannulation by PowerLab (ADInstruments, Colorado Spring, CO). The blood samples were used to measure blood biochemistry using CG4 and CHEM8 cartridges by iSTAT (Abbott, Washington, DC), complete blood count (WBC, RBC, platelet, and hematocrit) by ADVIA 120 Hematology System (SIEMENS Healthineers, Erlangen, Germany), and hemostasis (prothrombin time (PT), fibrinogen) by Start-4 (Diagnostic Stago Inc., Parsippany, NJ). The tissues were taken immediately after euthanasia to extract RNA to measure gene expression of HIF-1α, Glut1 (glucose transporter protein type 1), VEGFa (vascular endothelial growth factor A), Hexokinase, and EPO (erythropoietin) by PCR using a thermal cycler (Bio-Rad, Hercules, CA); nuclear proteins were also extracted to measure tissue levels of HIF-1α The lung tissue taken at 4 h was weighed as wet weight, and its dry weight was measured after drying in the oven at 60 °C for 10–14 days until there was no weight change for 3 consecutive days. The wet/dry weight ratio was then calculated.

### Reverse transcription polymerase chain reaction (RT-PCR)

RNA was extracted from tissues as described previously^67^. cDNA was synthesized with M-MLV reverse transcriptase and random primers (Promega, Madison, WI) from 1 µg of total RNA. Amplification reaction mixtures (16 µl) consisted of 1 × iTaq Universal SYBR Green Supermix (Bio-Rad), 10 pmol each of forward and reverse primers, and 40 ng of cDNA in duplicate or triplicate technical replicates. Thermocycling parameters were 95 °C for 2 min followed by 40 cycles of 95 °C for 15 s and 60 °C for 30 s in a CFX96 thermocycler (Bio-Rad). Raw fluorescence values were analyzed and amplicon efficiencies calculated using LinRegPCR (v2021.1)^68,69^ with the PavrgECt method for efficiency averaging^70^. Target gene transcript abundance was normalized to the mean of two or three of the most stably expressed reference genes which were identified using the geNorm and Normfinder algorithms^71,72^. Rpl19, Polr2f, and B2m served as reference genes, for lung, muscle, and kidney tissues, while B2m and Polr2f served as reference genes for liver samples. Relative gene expression was calculated as described previously^73^.

### ELISA

Tissue samples were weighed to 10 mg, minced with a razor blade, homogenized with a Kimble Pellet Pestle Cordless Motor (Millville, NJ), and sonicated using an Omni Ruptor 250 Ultrasonic Homogenizer (Vernon Hills, IL). Nuclear protein was extracted from rat lung, kidney, and liver tissue samples using a Nuclear Extraction Kit (Abcam, Waltham, MA). For HIF-1α analysis, tissue samples were normalized by protein content such that when loading 100 µL of sample per well, 1 µg of equivalent nuclear protein was loaded. HIF-1α levels were measured using a sandwich ELISA kit (LSBio, Seattle, WA) following manufacturer protocols.

### Study approval

This animal study was conducted under the protocol approved by the Institutional Animal Care and Use Committee of the U.S. Army Institute of Surgical Research and in compliance with the Animal Welfare Act, the implemented Animal Welfare Regulations, and the principles of the “Guide for the Care and Use of Laboratory Animals”. The current study was reported following the guidelines of ARRIVE (Animals in Research Reporting In Vivo Experiments, PLoS Bio 8(6), e1000412,2010).

### Data analysis

A sample size of 4 rats per group was used to perform a pilot proof-of-concept study to determine whether administration of MK-8617 prior to trauma mitigated lactate after polytrauma and hemorrhage. From that pilot study, the lactate at 4 h was 6.60 ± 3.83 mmol/l and 2.37 ± 0.73 mmol/l in rats treated with vehicle and MK-8617, respectively. Using a two-sample t-test with a pooled standard deviation of 2.76, a sample size of 7 rats was determined for post-trauma intravenous administration of MK-8617 or vehicle in the polytrauma and hemorrhage model to meet our expectations of 2-sided test 95% confidence intervals and 80% power in reducing the lactate level at 4 h by treatment with MK-8617 versus vehicle. For the lethal hemorrhagic shock model, the primary goal was to determine whether administration of PHDi extended survivability from a range of 0–20% to a range of 90–100% at 60 min after hemorrhage. A sample size of 13 rats per treatment group was initially estimated to provide 80% power to observe a statistical improvement in survivability from 20 to 80% between the vehicle and MK-8617 (low dose) groups. Based on the preliminary data from the lower dose of MK-8617 (0.5 mg) compared to the vehicle group (n = 13 per group) with survival proportions of 0.15 and 0.62 in 60-min follow-up and 0.08 and 0.46 in 240-min follow-up, the sample sizes calculated were 14 and 16 rats for 60- and 240-min follow-up, respectively. Based on these parameters, a sample size of 16 rats per treatment group (vehicle, low-dose MK-8617, and high-dose MK-8617) was determined to power sufficiently for the expectations of 95% confidence intervals and 80% power. A randomization process was used to allocate animals to the treatment groups. The data analysis was performed by GraphPad Prism 9 (GraphPad Software, San Diego, CA). The two-way repeated ANOVA (parametric) was used to test mean differences among the groups or within each group for the continuous variables over multiple study time points, followed by a pairwise comparison with the Tukey method if applicable. The time to death was recorded, and survivability was analyzed by both chi-square or Fisher exact tests and Kaplan–Meier plots with log-rank tests. Data are presented as means ± standard deviation, and statistical significance is accepted at p < 0.05.

### Supplementary Information

Supplementary Information.

## Data Availability

The datasets generated during and/or analyzed during the current study are available from the corresponding author on reasonable request.
