# Supplementary material for: Prolyl hydroxylase domain inhibitor is an effective pre-hospital pharmaceutical intervention for trauma and hemorrhagic shock
Source: Sci Rep. 2024 Feb 16;14:3874. doi: 10.1038/s41598-024-53945-w (PMC10873291; doi:10.1038/s41598-024-53945-w)
Supplement: Supplementary file 1 — Supplementary Information. [file 41598_2024_53945_MOESM1_ESM.pdf]

Figure 1S:

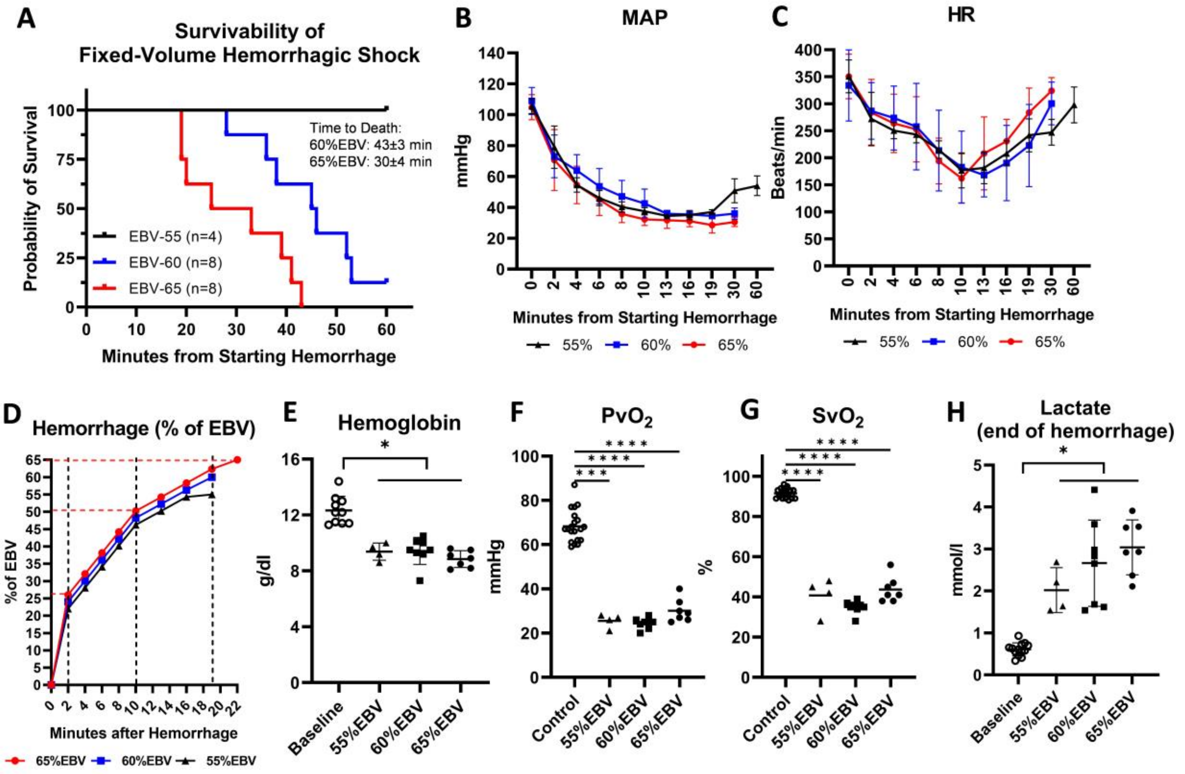

Fig.S1. Lethal hemorrhagic shock model: (A) Kaplan-Meier analysis of rats with 55%, 60% and 65% estimated blood volume (EBV) hemorrhage; (B) Mean arterial blood pressure (MAP) and (C) heart rate (HR) within an hour after hemorrhage; (D) Estimated percentage of blood loss during sequential blood withdrawal. Anemic and ischemic hypoxic response at the end of hemorrhage: (E) Hemoglobin; (F) Venous Oxygen Pressure (PvO_2); (G) Venous Oxygen Saturation (SvO2); and (H) Lactate. *: $p < 0.05$; **: $p < 0.01$; ***: $p < 0 .001$; ****: $p < 0.0001$.

Figure 2S:

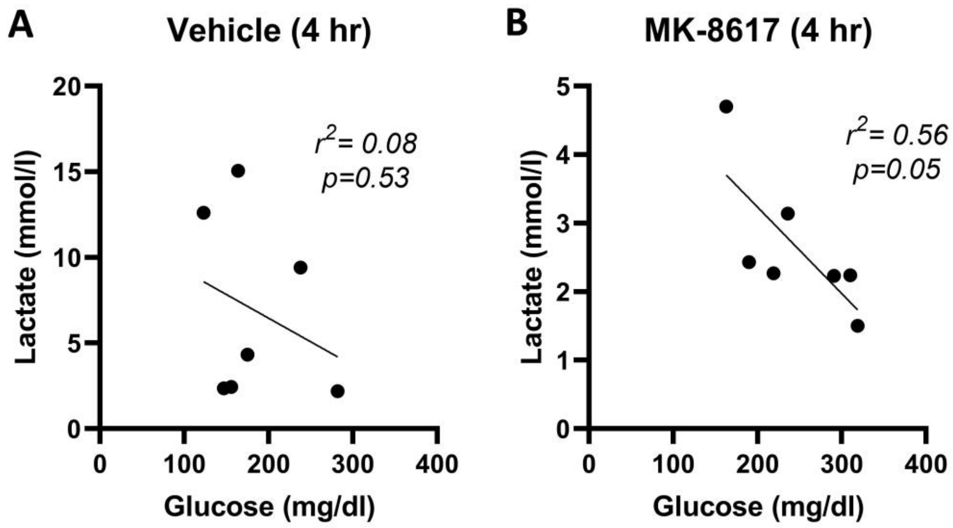

Fig. S2. Correlation of glucose and lactate 4 hr after polytrauma and hemorrhage. (A) Vehicle-treated animals showed no significant correlation. (B) MK-8617-treatedrats had a significant negative correlation between glucose and lactate, suggesting that lactate recycling is occurring through gluconeogenesis. r^2: coefficient of determination.

Figure 3S:

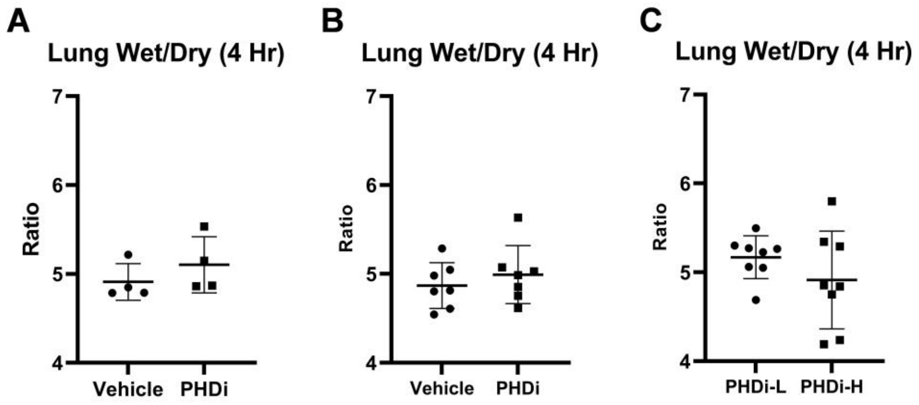

Fig. S3. Lung wet/dry weight ratio at 4 hr after polytrauma/hemorrhage or lethal hemorrhage. (A) Lungs from rats treated with vehicle or MK-8617 by gavage prior to trauma in polytrauma/hemorrhage model had similar wet/dry ratios. (B) Lungs from rats treated with vehicle or MK-8617 at 20 min after trauma in the polytrauma/hemorrhage model also had similar wet/dry ratios. (C) Lungs from rats treated with low or high dose of MK-8617 at 20 min after hemorrhage in the lethal hemorrhagic shock model.

Table 1S:

	Vehicle			MK-8617		
	BL	120 min	240 min	BL	120 min	240 min
pH	7.30±0.06	7.22±0.09	7.26±0.14	7.29±0.04	7.28±0.02	7.34±0.02
PCO2 (mmHg)	67.8±8.9	71.6±12.1	61.7±12.6	73.1±4.1	65.7±3.3	56.4±3.7
PO2 (mmHg)	71.5±4.2	39.5±2.1	33.3±4.2	65.8±13.3	34.5±4.8	27.0±4.6
Beecf (mmol/L)	6.8±1.5	1.3±4.5	-1.0±7.0	5.0±4.1	4.3±1.0	4.8±1.9
HCO3 (mmol/L)	33.2±0.9	29.0±3.3	27.2±4.2	32.6±1.0	30.7±0.9	30.5±1.9
TCO2 (mmol/L)	35.1±1.0	31.0±3.4	29.7±4.9	34.8±1.0	32.8±1.0	32.0±2.2
SatO2 (%)	88.0±7.0	52.1±14.2	39.5±16.4	90.0±1.8	65.5±4.7	58.0±7.8
Lactate (mmol/L)	0.54±0.07	5.25±2.55	6.60±3.83	0.49±0.07	2.32±0.32	2.37±0.73
Na (mmol/L)	156±1.0	152±3	155±1.0	157±1.0	153±3	156±2
K (mmol/L)	2.80±0.14	4.53±0.35	5.38±0.57	2.93±0.15	4.63±0.10	5.03±0.39
CL (mmol/L)	100±1.0	99±1.0	104±2	101±1	99±1.0	103±0
Glucose (mg/dl)	162±16	303±53	96±59	163±10	308±25	151±29
BUN (mg/dl)	12.3±1.0	23.0±1.6	39.8±6.6	11.3±0.5	23.3±1.5	33.3±3.0
Creatinine (mg/dl)	0.28±0.10	0.73±0.05	1.15±0.18	0.33±0.05	0.75±0.06	1.10±0.08
AnGap	29.3±1.5	30.0±3.4	31.7±2.3	29.3±1.5	29.8±1.5	30.3±1.5

Table S1. Biochemistry of rats treated with vehicle or MK-8617 by gavage prior to trauma. The biochemistry measurements were collected by iSTAT (CG4+ and CHEM8+ cartridges) in whole blood collected from the femoral vein at baseline (BL), 120 min and 240 min after trauma.

Table 2S:

	Vehicle				MK-8617			
	BL	60 min	120 min	240 min	BL	60 min	120 min	240 min
pH	7.31±0.0.	7.21±0.08	7.24±0.09	7.21±0.17	7.34±0.04	7.27±0.04	7.31±0.01	7.35±0.03
PCO2 (mmHg)	57.4±7.1	65.1±9.7	62.9±12.6	59.5±15.1	55.0±8.1	61.5±7.5	55.0±4.2	52.3±4.1
PO2 (mmHg)	74.3±9.4	41.8±5.2	42.8±5.6	35.0±5.7	75.1±8.1	49.9±3.9	49.4±4.9	41.7±3.9
Beecf (mmol/L)	2.7±2.3	-1.7±2.2	-0.9±2.9	-0.4±6.9	3.4±1.9	-1.1±2.3	1.3±1.5	3.4±1.3
HCO3 (mmol/L)	28.9±2.4	26.0±1.5	26.3±2.4	26.4±5.3	29.2±2.0	28.1±2.2	27.6±1.6	29.0±1.2
TCO2 (mmol/L)	30.7±2.6	27.9±1.6	28.3±2.7	28.0±5.2	30.7±2.4	30.0±2.5	29.1±1.7	30.6±1.4
SatO2 (%)	92.6±2.4	63.0±10.1	65.3±10.8	57.5±11.1	94.1±1.1	77.7±4.8	79.4±5.0	73.1±5.7
Lactate (mmol/L)	0.60±0.27	3.42±1.00	4.25±1.85	7.05±5.27	0.61±0.26	2.53±0.76	2.63±0.60	2.64±1.03
Na (mmol/L)	166±6	162±2	162±6	171±5	168±5	161±7	166±4	170±4
K (mmol/L)	2.96±0.44	3.71±0.59	4.09±0.78	5.07±1.91	3.09±0.29	3.77±0.68	3.90±0.57	4.27±0.61
CL (mmol/L)	114±5	112±2	111±5	119±4	116±4	110±5	113±3	116±3
Glucose (mg/dl)	174±46	338±69	362±75	212±87	169±25	327±73	334±82	228±81
BUN (mg/dl)	9.4±1.5	15.4±3.3	19.2±2.6	26.0±3.5	9.4±3.2	15.3±4.8	17.9±4.7	26.1±5.1
Creatinine (mg/dl)	0.20±0	0.34±0.05	0.49±0.07	0.74±0.17	0.20±0	0.37±0.08	0.41±0.14	0.66±0.22
AnGap	28.7±3.5	29.1±2.0	29.9±2.8	33.5±4.0	29.1±4.9	29.3±4.7	30.3±1.8	31.0±4.1

Table S2. Biochemistry of rats treated with vehicle or MK-8617 intravenously at 20 min after trauma. The biochemistry measurements were collected by iSTAT (CG4+ and CHEM8+

cartridges) in whole blood collected from the femoral vein at baseline (BL), 60 min, 120 min and 240 min after trauma.

Table 3S:

	Vehicle			MK-L			MK-H		
	BL	60 min	240 min	BL	60 min	240 min	BL	60 min	240 min
pH	7.27±0.12	6.96±0.05	7.16±0.06	7.28±0.13	7.04±0.05	7.27±0.21	7.32±0.03	7.20±0.08	7.38±0.04
PCO2 (mmHg)	63.1±17.1	84.3±11.8	54.0±0.4	62.2±23.2	65.5±8.4	52.4±12.5	53.7±5.8	53.5±5.7	46.3±5.5
PO2 (mmHg)	71.4±9.1	29.7±5.0	30.5±2.1	70.9±8.5	36.5±6.0	34.8±6.3	74.3±13.7	36.6±5.2	47.2±5.5
Beecf (mmol/L)	1.7±4.1	-15.0±1.4	-9.5±3.5	1.8±3.1	-13.3±3.0	-3.6±9.4	1.9±1.5	-7.1±4.0	2.2±2.4
HCO3 (mmol/L)	28.4±2.8	17.4±1.8	19.1±2.3	28.2±2.3	16.9±2.3	23.3±5.9	28.1±1.6	20.9±2.7	27.2±2.0
TCO2 (mmol/L)	30.1±2.7	20.0±2.8	20.5±2.1	29.9±2.3	18.9±2.7	24.9±5.6	29.6±1.6	22.4±2.7	28.7±2
SatO2 (%)	91.8±2.9	28.5±13.4	42.0±1.4	91.9±2.1	52.1±19.5	66.0±20.9	90.8±6.9	56.1±13.4	80.4±6.2
Lactate (mmol/L)	0.59±0.16	10.78±1.36	6.74±1.07	0.61±0.26	8.80±1.89	4.72±3.64	0.57±0.28	5.56±1.99	1.87±0.83
Na (mmol/L)	164±16		174	167±13		173±3	170±7		173±2
K (mmol/L)	3.15±0.49		4.2	2.97±0.39		4.78±1.25	2.99±0.29		3.73±0.24
CL (mmol/L)	111±10		122	113±9		121±4	116±5		119±2
Glucose (mg/dl)	169±21		80±1	176±33		116±53	184±32		179±43
BUN (mg/dl)	13.4±2.4		26	12.1±1.9		32.9±3.5	10.4±1.8		26.1±4.8
Creatinine (mg/dl)	0.24±0.07		0.9	0.23±0.07		0.92±0.14	0.21±0.03		0.51±0.29
AnGap	29.4±1.5		36	31.6±4.2		34.3±2.4	31.5±3.1		32.6±1.4

Table S3. Biochemistry of rats treated with vehicle, low or high dose of MK-8617 (PHDi-L or PHDi-H) intravenously at 20 min after hemorrhage. The biochemistry measurements were collected by iSTAT (CG4+ and CHEM8+ cartridges) in whole blood collected from the femoral vein at baseline (BL), 60 min prior to whole blood resuscitation (CG4 only), and 240 min (180 min after whole blood resuscitation).

Table 4S:

	Vehicle			MK-L			MK-H		
	BL	Last Hem	240 min	BL	Last Hem	240 min	BL	Last Hem	240 min
WBC ($x10^3/\mu l$)	7.48±2.17	2.42±0.74	10.69±0.47	7.74±1.90	2.72±0.73	13.50±4.79	7.74±2.48	3.23±0.84	13.63±3.67
RBC ($x10^6/\mu l$)	6.34±0.55	4.26±0.39	6.01±0.35	6.50±0.49	4.64±0.47	6.05±0.99	6.46±0.38	4.74±0.37	5.24±0.53
Hb (g/dl)	12.5±1.0	8.6±0.8	12.0±0.1	12.7±0.8	9.2±0.8	11.9±1.7	13.1±1.0	9.4±0.8	10.6±1.0
Platelet ($x10^6/\mu l$)	833±144	654±143	785±48	860±115	706±106	808±163	889±97	744±81	789±124
HCT (%)	36.1±2.7	24.6±2.3	36.2±2.6	36.8±2.0	26.5±2.2	35.0±7.1	36.8±2.1	27.5±1.9	29.8±2.9

Table S4. Complete blood count (CBC) of rats treated with vehicle, low or high dose of MK-8617 (PHDi-L or PHDi-H) intravenously at 20 min after hemorrhage. The CBC was measured in whole blood collected from the femoral vein at baseline (BL), end of hemorrhage (last Hem), and 240 min (180 min after whole blood resuscitation).

Table 5S:

Primer	Forward Primer Sequence (5' → 3')	Reverse Primer Sequence (5' → 3')	Reference
Gapdh	CCGCATCTTCTTGTGCAGTG	GGTAACCAGGCGTCCGATAC	This study
Hprt	GACCGGTTCTGTCATGTCG	ACCTGGTTCATCATCACTAATCAC	[11]
Actb	CAACCTTCTTGCAGCTCCTC	AGGGTCAGGATGCCTCTCTT	[12]
Tbp	TTCTGGGAAAATGGTGTGC	CCCACCATGTTCTGGATCTT	[13]
Rpl19	ACCTGGATGCGAAGGATGAG	CCATGAGAATCCGCTTGTTT	[14]
Tfrc	GTTGTTGAGGCAGACCTTCA	ATGACTGAGATGGCGGAAAC	This study
B2m	GTCGTGCTTGCCATTCAGA	ATTTGAGGTGGGTGGAACTG	[15]
Polr2f	AAAATGCTGAGGAGGAGGGTC	TGGAGTGGTGATACGCTTCTG	This study
Egf	ACCAACACGGAGGGAGGCTACAA	GCGGTCCACGGATTCAACATACA	[16]
Hif1a	AAGTCAGCAACGTGGAAGGT	CGTCATAGGCGGTTTCTTGT	[17]
Vegfa	AAGTGACCACATTCACTGTGAGCCT	GCTCACCGCCTTGGCTTGTCA	[18]
Hepc	GGCAGAAAGCAAGACTGATGAC	ACAGGAATAAATAATGGGGCG	[19]
Epo	GAATTGATGTCGCCTCCAGA	TGGAGTAGACCCGGAAGAGCT	[20]
HK	TCTGGGCTTCACCTTCTCAT	ATCAAGATTCCACAGTCCAGGT	[11]

1 Zhong HSimons, JW. Direct comparison of GAPDH, beta-actin, cyclophilin, and 28S rRNA as internal standards for quantifying RNA levels under hypoxia. Biochem Biophys Res Commun. 1999; 259: 523-526.
2 Foldager CB, Munir, S, Ulrik-Vinther, M, Soballe, K, Bunger, CLind, M. Validation of suitable house keeping genes for hypoxia-cultured human chondrocytes. BMC Mol Biol. 2009; 10: 94.
3 Zhang JY, Zhang, F, Hong, CQ, Giuliano, AE, Cui, XJ, Zhou, GJ, Zhang, GJCui, YK. Critical protein GAPDH and its regulatory mechanisms in cancer cells. Cancer Biol Med. 2015; 12: 10-22.
4 Muraoka WT, Granados, JC, Gomez, BI, Nicholson, SE, Chung, KK, Shupp, JW, Bynum, JA, Dubick, MABurmeister, DM. Burn resuscitation strategy influences the gut microbiota-liver axis in swine. Sci Rep. 2020; 10: 15655.
5 Ramakers C, Ruijter, JM, Deprez, RHMoorman, AF. Assumption-free analysis of quantitative real-time polymerase chain reaction (PCR) data. Neurosci Lett. 2003; 339: 62-66.
6 Ruijter JM, Ramakers, C, Hoogaars, WM, Karlen, Y, Bakker, O, van den Hoff, MJMoorman, AF. Amplification efficiency: linking baseline and bias in the analysis of quantitative PCR data. Nucleic Acids Res. 2009; 37: e45.
7 Karlen Y, McNair, A, Perseguers, S, Mazza, CMermod, N. Statistical significance of quantitative PCR. BMC Bioinformatics. 2007; 8: 131.
8 Vandesompele J, De Preter, K, Pattyn, F, Poppe, B, Van Roy, N, De Paepe, ASpeleman, F. Accurate normalization of real-time quantitative RT-PCR data by geometric averaging of multiple internal control genes. Genome Biol. 2002; 3: RESEARCH0034.
9 Andersen CL, Jensen, JLOrntoft, TF. Normalization of real-time quantitative reverse transcription-PCR data: a model-based variance estimation approach to identify genes suited for normalization, applied to bladder and colon cancer data sets. Cancer Res. 2004; 64: 5245-5250.
10 Pfaffl MW. A new mathematical model for relative quantification in real-time RT-PCR. Nucleic Acids Res. 2001; 29: e45.
11 Waskova-Arnostova P, Kasparova, D, Elsnicova, B, Novotny, J, Neckar, J, Kolar, FZurmanova, J. Chronic hypoxia enhances expression and activity of mitochondrial creatine kinase and hexokinase in the rat ventricular myocardium. Cell Physiol Biochem. 2014; 33: 310-320.
12 Soultanova A, Mikulski, Z, Pfeil, U, Grau, VKummer, W. Calcitonin Peptide Family Members Are Differentially Regulated by LPS and Inhibit Functions of Rat Alveolar NR8383 Macrophages. PLoS One. 2016; 11: e0163483.
13 Tan SC, Carr, CA, Yeoh, KK, Schofield, CJ, Davies, KEClarke, K. Identification of valid housekeeping genes for quantitative RT-PCR analysis of cardiosphere-derived cells preconditioned under hypoxia or with prolyl-4-hydroxylase inhibitors. Mol Biol Rep. 2012; 39: 4857-4867.
14 Zhou L, Lim, QE, Wan, GToo, HP. Normalization with genes encoding ribosomal proteins but not GAPDH provides an accurate quantification of gene expressions in neuronal differentiation of PC12 cells. BMC Genomics. 2010; 11: 75.
15 Yurube T, Takada, T, Hirata, H, Kakutani, K, Maeno, K, Zhang, Z, Yamamoto, J, Doita, M, Kurosaka, MNishida, K. Modified house-keeping gene expression in a rat tail compression loading-induced disc degeneration model. J Orthop Res. 2011; 29: 1284-1290.
16 Deming L, Ziwei, L, Xueqiang, GCunshuan, X. Restoration of CpG Methylation in The Egf Promoter Region during Rat Liver Regeneration. Cell J. 2015; 17: 576-581.
17 Giusti B, Marini, M, Rossi, L, Lapini, I, Magi, A, Capalbo, A, Lapalombella, R, di Tullio, S, Samaja, M, Esposito, F et al. Gene expression profile of rat left ventricles reveals persisting changes following chronic mild exercise protocol: implications for cardioprotection. BMC Genomics. 2009; 10: 342.
18 Han L, Li, J, Chen, Y, Zhang, M, Qian, L, Chen, Y, Wu, Z, Xu, YLi, J. Human Urinary Kallidinogenase Promotes Angiogenesis and Cerebral Perfusion in Experimental Stroke. PLoS One. 2015; 10: e0134543.
19 Merle U, Fein, E, Gehrke, SG, Stremmel, WKulaksiz, H. The iron regulatory peptide hepcidin is expressed in the heart and regulated by hypoxia and inflammation. Endocrinology. 2007; 148: 2663-2668.
20 Kletkiewicz H, Hyjek, M, Jaworski, K, Nowakowska, ARogalska, J. Activation of hypoxia-inducible factor-1alpha in rat brain after perinatal anoxia: role of body temperature. Int J Hyperthermia. 2018; 34: 824-833.

Table S5. List of the primers used in qPCR.
